# An NMR-Based Metabolomic Approach to Unravel the Preventive Effect of Water-Soluble Extract from *Dendrobium officinale* Kimura & Migo on Streptozotocin-Induced Diabetes in Mice

**DOI:** 10.3390/molecules22091543

**Published:** 2017-09-15

**Authors:** Hong Zheng, Linlin Pan, Pengtao Xu, Jianjun Zhu, Ruohan Wang, Wenzong Zhu, Yongsheng Hu, Hongchang Gao

**Affiliations:** 1 Institute of Metabonomics & Medical NMR, School of Pharmaceutical Science, Wenzhou Medical University, Wenzhou 325035, China; 123zhenghong321@163.com (H.Z.); 15867750670@163.com (L.P.); xupt92@163.com (P.X.); wangruohan678@163.com (R.W.); yshu@outlook.com (Y.H.); 2 Wenzhou Academy of Agricultural Sciences, Wenzhou 325006, China; zjj7257@sohu.com; 3 Department of Neurology Rehabilitation, Wenzhou Chinese Medicine Hospital, Wenzhou 325000, China; gdkingzwz@126.com

**Keywords:** amino acid, diabetes, energy metabolism, glucose-lowering, liver

## Abstract

*Dendrobium officinale* Kimura & Migo (*D. officinale*) is a precious herbal medicine. In this study, we investigated metabolic mechanism underlying the effect of *D. officinale* water extract (DOWE) on diabetes prevention in mice after streptozotocin (STZ) exposure using NMR-based metabolomics. Interestingly, we found a decrease in blood glucose and an increase in liver glycogen in mice pretreated with DOWE after STZ exposure. The DOWE pretreatment significantly increased citrate and glutamine in the serum as well as creatine, alanine, leucine, isoleucine, valine, glutamine, glutathione and taurine in the liver of STZ-treated mice. Furthermore, serum glucose was significantly negatively correlated with citrate, pyruvate, alanine, isoleucine, histidine and glutamine in the serum as well as alanine and taurine in the liver. These findings suggest that the effect of DOWE on diabetes prevention may be linked to increases in liver glycogen and taurine as well as the up-regulation of energy and amino acid metabolism.

## 1. Introduction

Diabetes mellitus (DM), characterized by hyperglycemia due to impaired β-cell function or insulin resistance, is one of the most prevalent chronic metabolic diseases. A series of complications involving multiple organs can be caused by DM [1], affecting a growing number of people’s health around the world. The International Diabetes Federation (IDF) estimates that in 2015, 415 million people had diabetes worldwide, while approximately 193 million were undiagnosed [2]. If nothing is done, this number will increase to 642 million in 2040 [2]. Thus, it is of great interest and importance to develop a promising strategy for prevention and treatment of DM.

Treatment of diabetes using plant extracts has a long history and also shows a promising future [3]. Tan et al. [4] reported that momordicosides in bitter melon can increase fatty acid oxidation and glucose disposal in both insulin-sensitive and insulin-resistant mice during glucose tolerance tests. *Astragalus* polysaccharide can improve insulin sensitivity by inhibiting the expression of protein tyrosine phosphatase 1B (PTP1B), a potential therapeutic target of DM, in type 2 diabetic (T2D) rats [5]. Lu et al. [6] found that total flavonoids from *Litsea Coreana leve* ameliorated hyperglycemia, hyperlipoidemia and insulin resistance in T2D rats. The antidiabetic activity of *Annona muricata* aqueous extract may be attributed to its hypolipidaemic effect as well as its protective effect on pancreatic β-cells [7]. Numonov et al. [8] and Kim et al. [9] revealed that polyphenolic and flavonoid compositions from *Geranium collinum* root and *Epimedium koreanum* Nakai possessed the antidiabetic activity via inhibition of PTP1B and α-glucosidase. The antidiabetic mechanisms of polyphenols from plant extracts may be also by increasing glucagon-like peptide-1 (GLP1) and insulin signaling [10]. Phytogenic polyphenols including pentacyclic triterpenes and flavonoids have been found as glycogen phosphorylase inhibitors for glycaemic control in diabetes [11]. Moreover, the antidiabetic activities through different mechanisms were also reported in other plant extracts, such as tea [12], American ginseng [13], *Radix astragali* [14], *Piper longum* [15], *Cistus laurifolius* [16], *Cinnamomum zeylanicum* [17], *Mulberry* [18], *Ocimum basilicum* [19], and others. According to the literature search, therefore, we found that more attention has been paid to plant extracts in order to discover a new strategy for treating DM.

*Dendrobium officinale* Kimura & Migo (*D. officinale*) is commonly consumed as a functional food supplement or herbal medicine worldwide, especially in Asian countries, owing to its immunologic, antioxidative and anticarcinogenic activities [20,21,22,23] as well as its beneficial effect on colonic health [24]. In addition, its antidiabetic activity has also been reported by several researchers. For example, Hou et al. [25] reported that the protective effect of *D. officinale* polysaccharides on streptozotocin (STZ)-induced diabetic complications in rats may be attributed to its antioxidant activity. The crude polysaccharides extracted from *D. officinale* offered therapeutic potential against diabetic cardio-myopathy in STZ-treated mice by inhibiting oxidative stress, inflammation and cardiac fibrosis [26]. However, there are only a few studies focusing on the effect of *D. officinale* on diabetes prevention, so further exploring its action mechanisms will advance the evidence-based application in management of DM. Generally, *D. officinale* can be chewed or sipped by pouring boiling and hot water, just like tea. Therefore, we were curious to know whether *D. officinale* water extract (DOWE) can prevent diabetes development. Metabolomics, as one of omics techniques, attempts to analyze a comprehensive set of small-molecule metabolites in biological samples and examines their alterations under a particular condition, such as disease or drug intervention. Since the modernization of traditional Chinese medicine (TCM) is becoming necessary and urgent [27], metabolomics as one of modern technologies has shown a great potential toward understanding the efficacy and mechanism of TCM [28,29]. In the field of metabolomics, nuclear magnetic resonance (NMR) spectroscopy is an attractive analytical method due to simple sample preparation, rapid analysis as well as high reproducibility. In previous studies, we have successfully used an NMR-based metabolomic approach to elucidate possible metabolic mechanisms of diabetic nephropathy [30], diabetic encephalopathy [31,32] as well as drug treatment [33]. In the present study, therefore, we analyzed metabolic profiles in the serum and liver of mice pretreated with or without DOWE after STZ exposure using an NMR-based metabolomic approach and aimed to explore potential metabolic mechanisms of DOWE on the prevention of DM.

## 2. Results 

### 2.1. The Main Chemical Compositions of Dendrobium officinale Water Extract

**Figure 1:**
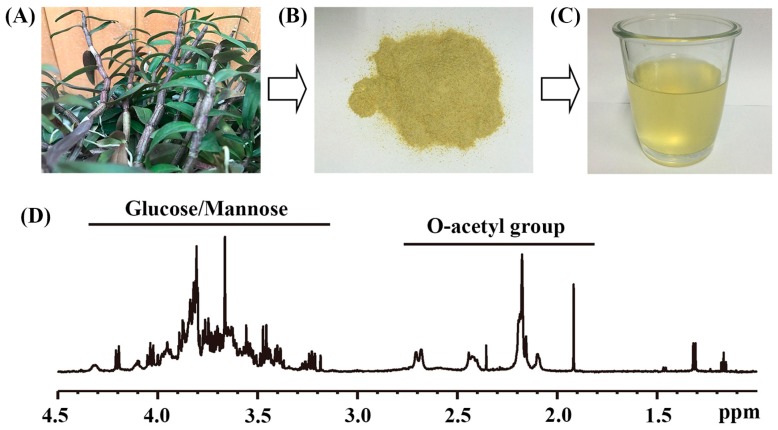
*Dendrobium officinale* water extraction: (**A**) photo of fresh *D. officinale* plants; (**B**) photo of stem powder; (**C**) photo of water extract solution; (**D**) a typical ^1^H-NMR spectrum of *D. officinale* water extract.

Figure 1A shows photos of fresh *D. officinale* plants, stem powder as well as its aqueous extract. The main chemical compositions of *D. officinale* water extract (DOWE) were analyzed using NMR spectroscopy and its ^1^H-NMR spectrum is shown in Figure 1B. It can be seen that DOWE mainly contain *O*-acetyl group and glucose/mannose. The yield of dried water extract was about 42.57 ± 4.45%, containing approximately 0.90 ± 0.05 mM/g *O*-acetyl group and 2.90 ± 0.20 mM/g glucose/mannose (Table 1). In this study, low-dose and high-dose water extracts (LWE and HWE) were prepared for mice pretreatment at 13.50 ± 1.50 and 27.00 ± 1.50 mM/L of *O*-acetyl group as well as 43.50 ± 4.50 and 87.00 ± 6.00 mM/L of glucose/mannose, respectively.

### 2.2. Potential Effect of DOWE on Diabetes Prevention in Mice

**Figure 2:**
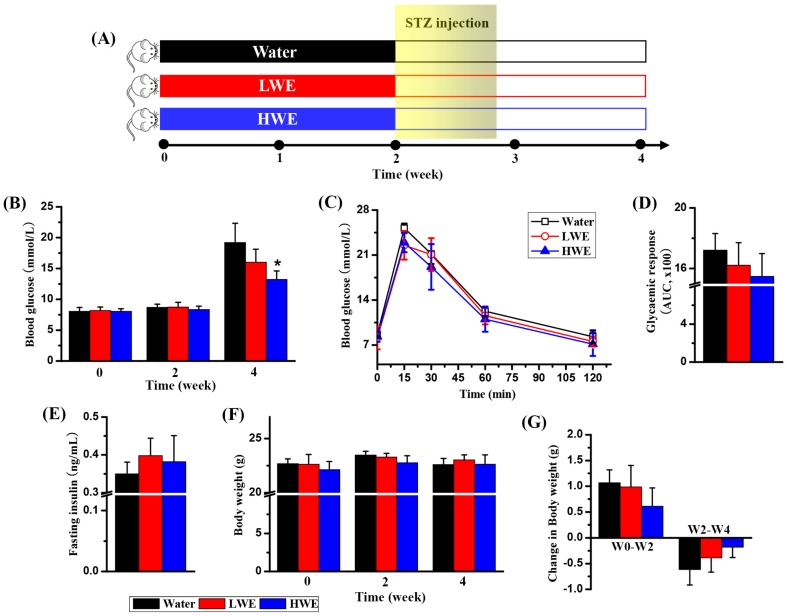
Changes in blood glucose level and body weight of STZ-treated mice after administration of *Dendrobium officinale* water extract: (**A**) experimental procedure; (**B**) blood glucose levels at 0, 2 and 4 weeks; (**C**) oral glucose tolerance test (OGTT) at 4 weeks; (**D**) area under the curve (AUC) of OGTT at 4 weeks; (**E**) fasting insulin level at 4 weeks; (**F**) body weight at 0, 2 and 4 weeks; (**G**) changes in body weight between 0 and 2 weeks (W0–W2) as well as between 2 and 4 weeks (W2–W4). Treatment: Water, distilled water; LWE, low-dose water extract; HWE, high-dose water extract. Significant level: * *p* < 0.05.

In this study, mice were randomly assigned to be pretreated with water, LWE or HWE for 2 weeks, and then continually exposed to low-dose STZ for 5 days after 2 weeks, as shown in Figure 2A. Figure 2B shows that random blood glucose level was no significant difference among these three groups at 0 and 2 weeks. However, interestingly, a significant decrease in random blood glucose level was detected in the HWE group relative to the water group (Figure 2B, *p* < 0.05). The LWE group also had a reduction in random blood glucose level, but no statistically significant difference, as compared with the water group. At 4 weeks, these three groups showed comparable glucose tolerance curves (Figure 2C), whereas glucose intolerance was slightly but not significantly decreased in mice pretreated with LWE and HWE than mice in the water group (Figure 2D).

Figure 2E shows that the LWE and HWE groups had an increase in fasting serum insulin level but no significant difference, compared with the water group. Body weight was not significantly varied among these three groups at 0, 2 and 4 weeks, as illustrated in Figure 2F. Furthermore, we calculated the change of body weight before and after STZ exposure. Before STZ exposure, body weight was increased in all three groups, whereas mice pretreated with HWE had a relatively low increase (Figure 2G). In addition, as can be seen from Figure 2G, STZ exposure can cause decreased body weight, but the reduction was relatively lower in the LWE and HWE groups than the water group.

### 2.3. Metabolic Response to DOWE in the Serum of STZ-Treated Mice

**Figure 3:**
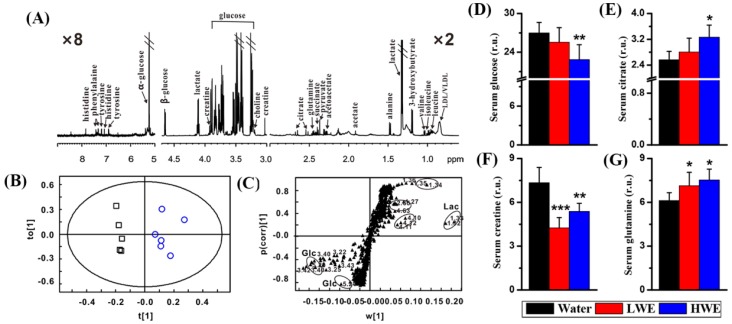
NMR-based serum metabolomic analysis: (**A**) a typical ^1^H-NMR spectrum from the serum of a healthy mouse; (**B**) PLS-DA score plot between the water group (○) and the HWE group (□); (**C**) PLS-DA loading plot (Lac, lactate; Glc, glucose); (**D**) serum glucose level; (**E**) serum citrate level; (**F**) serum creatine level; (**G**) serum glutamine level. Treatment: Water, distilled water; LWE, low-dose water extract; HWE, high-dose water extract. r.u.: relative unit. Significant level: * *p* < 0.05; ** *p* < 0.01; *** *p* < 0.001.

The pretreatment effect of DOWE on the serum metabolome in mice after STZ exposure was studied using an NMR-based metabolomic approach. Figure 3A shows a typical ^1^H-NMR spectrum obtained from the serum of a healthy mouse, where we identified a series of serum metabolites, involving energy metabolism (citrate, creatine, succinate, glucose, lactate and pyruvate), lipid metabolism (acetate, choline and LDL/VLDL), amino acid metabolism (alanine, glutamine, histidine, isoleucine, leucine, phenylalanine, tyrosine and valine) as well as ketone body metabolism (acetoacetate and 3-hydroxybutyrate). Then, PLS-DA was used to identify serum metabolic differences between the water group and the LWE group as well as between the water group and the HWE group, and the performance parameters of PLS-DA were listed in Table 2. PLS-DA between the water group and the HWE group revealed a good model performance (R^2^Y = 0.91, Q^2^ = 0.62), but not for the model between the water group and the LWE group (R^2^Y = 0.76, Q^2^ = 0.10). Therefore, the score and loading plots of PLS-DA between the water group and the HWE group were further shown in Figure 3B,C, respectively. According to Figure 3C, we found that lactate and glucose were mainly contributed to the separation between the water group and the HWE group in the score plot.

Furthermore, using univariate analysis, a significant decrease in serum glucose was observed in the HWE group relative to the water group (Figure 3D, *p* < 0.01), which is in agreement with the result from a handheld glucometer as shown in Figure 2B. Serum citrate was significantly increased in mice pretreated with HWE as compared with mice pretreated with water (Figure 3E). In addition, relative to mice in the water group, a significant decrease in serum creatine (Figure 3F) and a significant increase in serum glutamine (Figure 3G) were detected in mice pretreated with both LWE and HWE.

### 2.4. Metabolic Response to DOWE in the Liver of STZ-Treated Mice

**Figure 4:**
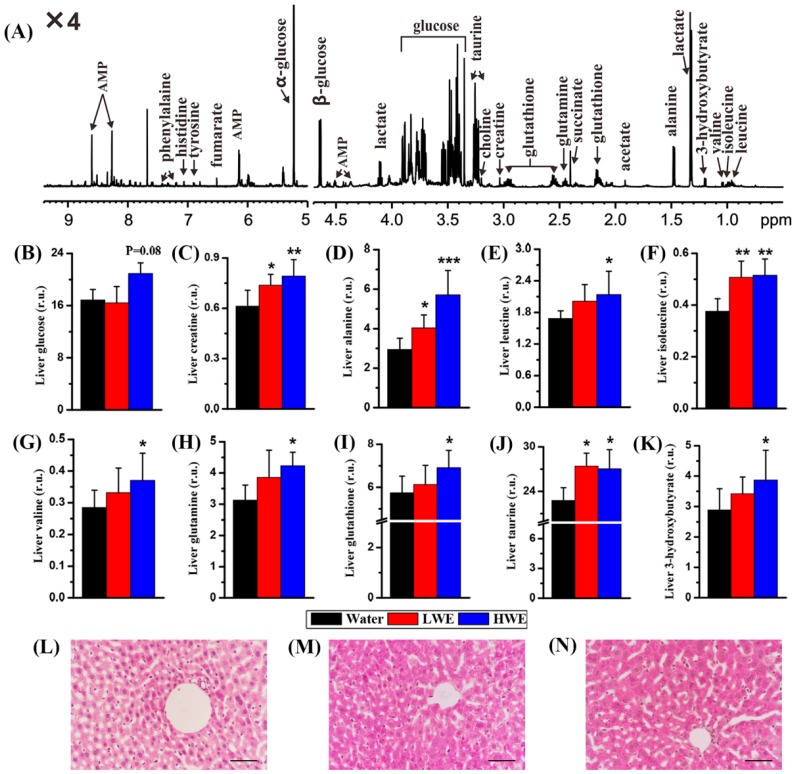
NMR-based liver metabolomic analysis: (**A**) a typical ^1^H-NMR spectrum from the liver of a healthy mouse; (**B**) liver glucose level; (**C**) liver creatine level; (**D**) liver alanine level; (**E**) liver leucine level; (**F**) liver isoleucine level; (**G**) liver valine level; (**H**) liver glutamine level; (**I**) liver glutathione level; (**J**) liver taurine level; (**K**) liver 3-hydroxybutyrate level; (**L**) periodic acid-Schiff (PAS) staining of the liver tissue in the water group; (**M**) PAS staining of the liver tissue in the LWE group; (**N**) PAS staining of the liver tissue in the HWE group. Treatment: Water, distilled water; LWE, low-dose water extract; HWE, high-dose water extract. Scale bar: 200 μm. r.u.: relative unit. Significant level: * *p* < 0.05; ** *p* < 0.01; *** *p* < 0.001.

A typical ^1^H-NMR spectrum from the liver of a healthy mouse is illustrated in Figure 4A. We identified a series of metabolites from NMR-based liver metabolome, such as 3-hydroxybutyrate, acetate, AMP, creatine, succinate, glucose, lactate, choline, alanine, glutathione, glutamine, histidine, isoleucine, leucine, phenylalanine, tyrosine, taurine, valine and fumarate. It can be seen from Table 2 that no reliable PLS-DA model based on liver metabolome can be developed between the water group and the LEW group (R^2^Y = 0.72, Q^2^ = −0.26) as well as between the water group and the HEW group (R^2^Y = 0.76, Q^2^ = 0.13).

Then, we evaluated the difference of metabolite levels among these three groups using univariate analysis. Contrary to serum glucose, Figure 4B shows that liver glucose level was slightly increased in the HWE group relative to the water group (*p* = 0.08). We also found that compared with mice pretreated with water creatine (Figure 4C), alanine (Figure 4D), isoleucine (Figure 4F) and taurine (Figure 4J) levels in the liver were significantly increased in mice pretreated with LWE and HWE. Moreover, mice pretreated with HWE had significantly higher levels of leucine (Figure 4E), valine (Figure 4G), glutamine (Figure 4H), glutathione (Figure 4I) and 3-hydroxybutyrate (Figure 4K) in the liver than mice pretreated with water. In this study, periodic acid-Schiff (PAS) staining was used to examine the change of liver glycogen among these three groups. Interestingly, we found that mice pretreated with LWE (Figure 4M) or HWE (Figure 4N) had an obviously increased hepatic glycogen level as compared with mice pretreated with water (Figure 4L).

### 2.5. Correlation Analysis between Serum Glucose and Metabolites in the Serum and Liver of Mice

**Figure 5:**
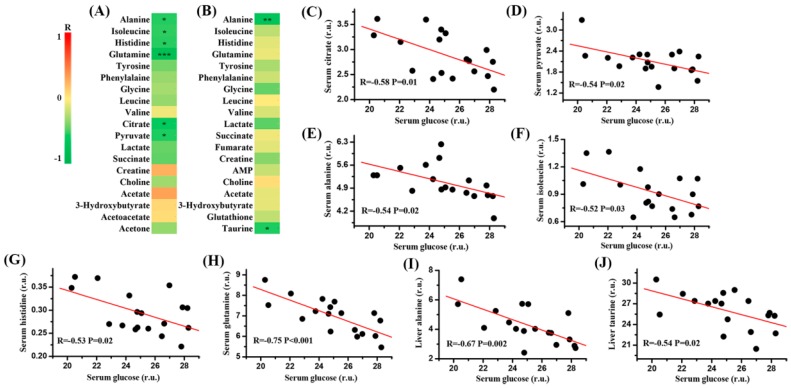
Correlation analysis: (**A**) correlation heatmap between serum glucose and other serum metabolites; (**B**) correlation heatmap between serum glucose and liver metabolites; (C) regression plot between serum glucose and serum citrate; (**D**) regression plot between serum glucose and serum pyruvate; (**E**) regression plot between serum glucose and serum alanine; (**F**) regression plot between serum glucose and serum isoleucine; (**G**) regression plot between serum glucose and serum histidine; (**H**) regression plot between serum glucose and serum glutamine; (**I**) regression plot between serum glucose and liver alanine; (**J**) regression plot between serum glucose and liver taurine. r.u.: relative unit. Significant level: * *p* < 0.05; ** *p* < 0.01; *** *p* < 0.001.

Results from correlation analysis between blood glucose and metabolites in the serum and liver of mice were illustrated in Figure 5A,B, respectively. We found that serum glucose was significantly negatively correlated with citrate (Figure 5C, R = −0.58, P = 0.01), pyruvate (Figure 5D, R = −0.54, P = 0.02), alanine (Figure 5E, R = −0.54, P = 0.02), isoleucine (Figure 5F, R = −0.52, P = 0.03), histidine (Figure 5G, R = −0.53, P = 0.02) and glutamine (Figure 5H, R = −0.75, P < 0.001) in the serum of mice. In addition, alanine (R = −0.67, P = 0.002) and taurine (R = −0.54, P = 0.02) in the liver were significantly negatively correlated with serum glucose, as shown in Figure 5I,J, respectively.

## 3. Discussion

In most Asian and Western countries, *D. officinale* is prepared by steeping in hot water and then the water-soluble constituents are consumed. Xing et al. [21] reported that *O*-acetylglucomannan as a water-soluble polysaccharide is the major active ingredient of *D. officinale*. Moreover, monosaccharides in *D. officinale* mainly include mannose and glucose [21]. In this study, NMR analysis also shows that *O*-acetyl group, mannose and glucose are the main constituents in *D. officinale* water extract (DOWE). The immunologic, antioxidative and anticarcinogenic activities of *D. officinale* have been reported [20,21,22,23], whereas its effect on diabetes prevention is still poorly understood. Therefore, we investigated metabolic mechanisms underlying the effect of DOWE on diabetes prevention in mice using an NMR-based metabolomic approach.

Interestingly, pretreatment of mice with high-dose water extract of *D. officinale* (HWE) can significantly decrease blood glucose level after STZ exposure. We also found that suppression of blood glucose level during OGTT was superior in mice after HWE pretreatment. Moreover, after STZ exposure, a significantly lower level of serum glucose was also detected in mice pretreated with HWE using a metabolomic method. These findings suggest that DOWE may possess a potential effect on diabetes prevention. Additionally, it is worth noting that DOWE can relatively reduce diabetes-induced weight loss. Contrary to blood glucose level, however, liver glucose was slightly increased in mice pretreated with HWE after STZ exposure. According to the PAS staining, an obviously increased hepatic glycogen level was also found in mice pretreated with DOWE as compared with the control mice. Glycogen is the main intracellular storable form of glucose in animals, which is associated with extracellular glucose level and insulin activity. Although glucose is one of main monosaccharides in DOWE, an increased hepatic glycogen level in mice pretreated with DOWE could not be simply attributed to a relatively high glucose intake. On the one hand, mice were subjected to DOWE during the first two weeks, but at two weeks mice fed DOWE and water had an almost equal level of blood glucose, indicating that mice possessed sufficient ability to metabolize extra glucose before STZ exposure. On the other hand, all mice were given to standard chow and tap water during the next two weeks, and hence further minimize the impact of glucose from DOWE. In addition, STZ can cause pancreatic β-cell destruction resulting in decreased glycogen synthesis in the liver [34]. A reduction of hepatic glycogen level was also reported in T2D patients relative to healthy individuals [35]. Our results indicate that the defective glycogen storage induced by diabetes may be partially corrected by DOWE intake. This phenomenon has also been reported in other plant extracts, such as *Zizyphus spina-christi* leaf [36], *Ficus amplissima* bark [37] and *Senna singueana* stem bark [38]. Thus, we speculate that DOWE may stimulate glycogenesis and increase storage of glucose as glycogen in the liver thereby decreasing blood glucose level. However, their detailed relationships need to be further explored.

Excepting glycogen synthesis, glucose is the primary energy substrate for maintaining cell functions. Glucose can be converted to pyruvate and then enter the TCA cycle for cellular energy supply. In this study, metabolomic results reveal that DOWE may increase energy metabolism as indicated by a significantly increase in serum citrate, as one of key TCA intermediates, in mice pretreated with HWE as compared with the control mice. Most interestingly, we found that serum glucose was significantly negatively correlated with two major energy-related metabolites, citrate and pyruvate. Creatine, mainly synthesized in the liver, is another important metabolite in energy homeostasis [39]. Compared with mice pretreated with water, liver creatine was significantly higher in mice after pretreatment of DOWE, indicating an increased energy metabolism. Yet, an opposite result was detected in the serum. Taken together, we suggest that the increase of energy metabolism may be contributed to reduced blood glucose level and diabetes prevention.

Amino acids play an essential role in human metabolism [40]. On the one hand, amino acids can be used as substrates for various metabolic pathways, such as TCA cycle, protein synthesis, gluconeogenesis, and others [41]. On the other, amino acids serve as regulators of metabolism, particularly for glucose metabolism, because some amino acids could stimulate a rise in insulin and glucagon concentrations [42]. In the present study, we found that serum glutamine as well as alanine, leucine, isoleucine, valine and glutamine in the liver were increased in mice pretreated with DOWE after STZ exposure, especially after HWE pretreatment, as compared with the control mice. Furthermore, it is worth noting that serum glucose was significantly negatively associated with alanine, isoleucine, histidine and glutamine in the serum as well as alanine in the liver. These results indicate that the glucose-lowering effect of DOWE may be implicated in the increase of amino acid metabolism. One explanation may be that amino acids accelerate the TCA cycle, thereby resulting in increased glucose consumption. In addition, some amino acids may stimulate insulin secretion and then reduce blood glucose level. In the present study, a slight increase in fasting insulin level was indeed detected in mice pretreated with DOWE; however, this phenomenon still needs to be further studied.

Taurine and glutathione as antioxidants can protect against oxidative stress in the liver [43,44]. Interestingly, we found that significantly increased levels of taurine and glutathione in the liver of mice pretreated with DOWE after STZ exposure relative to the control mice, especially after pretreatment of HWE. Haber et al. [43] reported that oxidative stress causes hyperglycemia-induced insulin resistance but taurine exerts a protective effect. A significantly negative relationship between serum glucose and liver taurine was observed in this study. Hence, we suggest that the effect of DOWE on diabetes prevention may be attributed to taurine-mediated defense against oxidative stress.

## 4. Materials and Methods

### 4.1. Animals

Male C57BL/6 mice (age = 8 weeks; body weight = 20.0 ± 2.0 g) were purchased from the SLAC Laboratory Animal Co. Ltd. (Shanghai, China) and housed in a specific pathogen-free (SPF) colony under a fully controlled condition (temperature = 23 ± 2 °C; humidity = 55 ± 5%; light:dark cycle = 12:12 h:h) at the Laboratory Animal Center of Wenzhou Medical University (Wenzhou, China). All mice were given free access to standard chow and tap water. This study was conducted in accordance to the Guide for the Care and Use of Laboratory Animals and approved by the Institutional Animal Care and Use Committee of Wenzhou Medical University (document number: wydw2016-0160).

### 4.2. Dendrobium officinale Water Extract (DOWE) Preparation

Fresh *D. officinale* stems were harvested from a local farm at Yueqing (Wenzhou, China). The plant samples were cut into small pieces and dried to constant weight at 65 °C. The dried samples were pulverized by a stainless steel pulverizer (Redsun Electromechanical Co. Ltd., Yongkang, China) and passed through a 40-mesh sieve (Figure 1A). Then 50 g powder was weighed and mixed with purified water (20 mL/g) in a flask. The mixture was extracted by heat reflux extraction at 90 °C for 3 h. After cooling, the mixture was centrifuged at 3000× *g* for 15 min at 4 °C. Subsequently, the supernatant was collected and the residue was subjected to re-extraction twice with 300 mL purified water. The extract was evaporated under reduced pressure at 55 °C for about 6 h, dried by lyophilization for 48 h and stored in drying vessel at room temperature until use. The dried water extract was weighed and the yield (%) was calculated on the basis of the dried weight of *D. officinale* powder.

The main components of the water extract were analyzed using a Bruker AVANCE III 600 MHz NMR spectrometer with a 5-mm TXI probe (Bruker BioSpin Gmbh, Rheinstetten, Germany) at 298 K. For NMR measurement, 300 μL of the extract was mixed with 300 μL of 99.5% D_2_O containing 0.05% sodium trimethlysilyl propionate-*d*_4_ (TSP), vortexed and centrifuged at 12,000× *g* at 4 °C for 15 min. Then, 500 μL of supernatant was transferred into a 5 mm NMR tube, and a standard single-pulse experiment with water signal pre-saturation (‘ZGPR’, Bruker BioSpin, Rheinstetten, Germany) was performed for NMR analysis. The main acquisition parameters included: acquisition time = 2.65 s/scan; data points = 64 K; relaxation delay = 2 s; spectral width = 12,000 Hz. NMR signals of DOWE were assigned in accordance to reported data [45]. The concentration of the main chemical composition was calculated according to its peak area relative to the TSP concentration.

### 4.3. DOWE Pretreatment and Streptozocin Exposure

The experimental procedure was illustrated in Figure 2A. After a 1-week-adaptation period, mice were randomly divided into three groups: (1) distilled water (*n* = 8; Water); (2) low-dose water extract (*n* = 8; LWE; 350 mg/kg body weight) and (3) high-dose water extract (*n* = 8; HWE; 700 mg/kg body weight). The water extract of *D. officinale* or its vehicle (distilled water) was administered intragastrically once daily at 9:00 a.m. for 2 weeks. After that, mice were treated with multiple low-dose streptozocin (STZ, Sigma-Aldrich, St. Louis, MO USA) after overnight fasting. The STZ solution was prepared in citrate buffer (0.5%, pH = 4.3) and given by intraperitoneal injection at dosage 40 mg/kg of body weight for 5 consecutive days according to the protocol of STZ-induced diabetic mice [46]. At 0, 2 and 4 weeks, blood glucose level was measured from a tail nick by a portable glucose monitor (ACCU-CHEK Active, Mannheim, Germany). Meanwhile, body weight was recorded with a digital balance (JY, Shanghai Minqiao Precise Science Co. Ltd., Shanghai, China). At 4 weeks, fasting insulin level was measured in the serum using an ELISA method (Merck, Darmstadt, Germany). Moreover, oral glucose tolerance test was conducted by measuring blood glucose from a tail nick at 0, 15, 30, 60 and 120 min after oral feeding with 40 mg glucose.

### 4.4. Serum and Liver Sample Preparation

All mice were sacrificed by decapitation at 4 weeks. Blood sample was centrifuged at 3000× *g* at 4 °C for 15 min, and the serum was collected and stored at −80 °C until use. In addition, liver sample was dissected immediately, frozen in liquid nitrogen, and stored at −80 °C until analysis. For metabolomic analysis, 200 μL of serum sample was thawed and mixed with 250 μL of phosphate buffer (0.2 mM Na_2_HPO_4_/NaH_2_PO_4_, pH = 7.4) and 50 μL of D_2_O. The diluted serum was vortexed and centrifuged at 12,000× *g* at 4 °C for 15 min. Subsequently, 500 μL of supernatant was transferred into a 5 mm NMR tube for NMR analysis. In addition, liver sample was extracted using the methanol-chloroform method described in our previous study [31]. Briefly, the frozen liver tissue was weighed into an Eppendorf tube and added with ice-cold methanol (4 mL/g) and distilled water (0.85 mL/g). The mixture was homogenized after thawing by a handheld homogenizer (KB3, QILINB-EIER, Haimen, China). Afterward, ice-cold chloroform (2 mL/g) and distilled water (2 mL/g) was consecutively added into the mixture and vortexed again. The mixture was allowed to stand on ice for 15 min and centrifuged at 10,000× *g* for 15 min at 4 °C. The supernatant was transferred to a new Eppendorf tube and lyophilized for 24 h. The lyophilized extract was redissolved in 500 μL of 99.5% D_2_O containing 0.05% TSP and transferred to a 5 mm NMR tube for metabolomic analysis.

### 4.5. Periodic Acid-Schiff Staining

Liver glycogen content was examined with periodic acid-Schiff (PAS) staining kit (Jiancheng Biotech, Nanjing, China) and the staining process was followed according to its instructions. In brief, liver sample was rapidly dissected, fixed overnight in 10% buffered neutral formalin, and then embedded in paraffin after ethanol washing. The paraffin-embedded liver tissue was sliced into 4 μm sections, deparaffinised in xylene and ethanol series, and washed in purified water. Subsequently, the paraffin section was incubated in periodic acid (reagent 1) for 8 min, washed in distilled water, and stained with Schiff reagent (reagent 2). Finally, the stained section was observed under a light microscope (Eclipse Ni, Nikon, Tokyo, Japan).

### 4.6. NMR-Based Metabolomic Analysis

^1^H-NMR spectra of serum and liver samples were recorded at 298 K using a Bruker AVANCE III 600 MHz NMR spectrometer with a 5-mm TXI probe. A ‘ZGPR’ pulse sequence (Bruker BioSpin, Gmbh, Rheinstetten, Germany) was used for liver samples in this study. However, the Carr-Purcell-Meiboom-Gill pulse sequence (‘CPMG’) with a fixed receiver-gain value was performed for serum samples to diminish broad peaks from protein and lipid signals. The main acquisition parameters were set as follows: acquisition time = 2.65 s/scan; data points = 64 K; relaxation delay = 2 s; spectral width = 12,000 Hz.

The NMR spectra were first preprocessed by auto-phase/baseline correction in the Topspin software (v2.1 pl4, Bruker Biospin, Rheinstetten, Germany). The spectra of serum samples were referenced to the methyl peak of lactate at 1.33 ppm, while the spectra of liver samples were referenced to TSP peak at 0 ppm. The ‘icoshift’ procedure in MATLAB (R2012a, The Mathworks Inc., Natick, MA, USA) was used to align NMR spectra [47]. The spectral region from 0.5 to 8.0 ppm without the residual water signals (from 4.4 to 5.2 ppm) were subdivided and integrated to spectral binning data with a size of 0.01 ppm for further multivariate analysis.

Partial least squares-discriminant analysis (PLS-DA) was used to examine the change in metabolic patterns between the two groups in SIMCA software (v12.0, Umetrics, Umeå, Sweden). Prior to PLS-DA, the spectral binning data were log-transformed and Pareto-scaled. A leave-one-out cross-validation (LOOCV) method was used for PLS-DA, where R^2^ and Q^2^ were calculated as the goodness of fit and the prediction of the model, respectively. These two parameters close to 1.0 indicate an excellent model.

NMR signals were assigned in accordance to published data for serum [48] and liver [49]. Moreover, 2D ^1^H-^1^H total correlation (TOCSY) and 2D ^13^C-^1^H heteronuclear single quantum coherence (HSQC) experiments were performed on representative samples to confirm uncertain identifications. The relative content of identified metabolite was calculated on the basis of its peak area relative to the TSP concentration.

### 4.7. Statistical Analysis

In this study, mice were randomly assigned to experimental procedures including housing and feeding, animal grouping, DOWE pretreatment as well as STZ exposure. NMR analysis was also conducted by masking the group labels. One-way analysis of variance (ANOVA) was performed for evaluating metabolic differences between different pretreatment groups using Student’s t test with Bonferroni correction in SAS 9.2 (SAS Institute Inc., Cary, NC, USA). Pearson’s correlation between serum glucose and metabolites in the serum and liver as well as the corresponding *p* value were analyzed by the MATLAB function (‘corrmatrix’, R2012a). Heatmap and linear regression plots were drawn in Origin software (v7.5, OriginLab, Northampton, MA, USA). A *p* value <0.05 was considered to be statistically significant.

## 5. Conclusions

In the present study, we found that blood glucose level can be reduced in mice pretreated with DOWE after STZ exposure, indicating its effect on diabetes prevention. Therefore, metabolic mechanisms underlying the effect of DOWE on diabetes prevention in mice were further explored using an NMR-based metabolomic approach. Results show that the glucose-lowering effect of DOWE may be implicated in the increase of liver glycogen synthesis, the up-regulation of energy and amino acid metabolism as well as taurine-mediated defense against oxidative stress. However, several limitations or further works should be considered: (1) dose- and time-response effects after intake of DOWE need to be further established; (2) it is of great interest to discover specific pharmacodynamic substances in DOWE; (3) these findings should be confirmed in other animal models prior to clinical trials; (4) key enzymes or proteins in metabolic pathways need to be explored for better understanding of the potential mechanisms of DOWE on diabetes prevention.

## Figures and Tables

**Table 1:** The concentrations of *O*-acetyl group and glucose/mannose in *D. officinale* water extract. **^a^**

Sample	Yield (%) ^b^	*O*-acetyl Group	Glucose/Mannose
Dried water extract	42.57 ± 4.45	0.90 ± 0.05 ^e^	2.90 ± 0.20
LWE ^c^	-	13.50 ± 1.50	43.50 ± 4.50
HWE ^d^	-	27.00 ± 1.50	87.00 ± 6.00

^a^ Data are presented as mean ± SE; ^b^ extract yield (%) was calculated as dried water extract (g)/100 g dried herbal powder; ^c^ low-dose water extract; ^d^ high-dose water extract; ^e^ the concentrations of *O*-acetyl group and glucose/mannose in dried water extract (mM/g) and HWE/LWE (mM/L) were calculated on the basis of TSP concentration.

**Table 2:** Performance parameters of PLS-DA using NMR-based serum and liver metabolomic data.

Sample	Model	PC ^a^	R^2 b^	Q^2 c^
Serum	Water ^**d**^ & LWE **^e^**	2	0.76	0.10
	Water & HWE **^f^**	2	0.91	0.62
Liver	Water & LWE	2	0.72	−0.26
	Water & HWE	2	0.76	0.13

**^a^** number of principal components; ^b^ goodness of fit; ^c^ prediction of the model; ^d^ mice fed water; ^e^ mice fed low-dose water extract; ^f^ mice fed high-dose water extract.

## Abbreviations

DM	Diabetes mellitus
DOWE	*Dendrobium officinale* water extract
HWE	High-dose water extract
LWE	Low-dose water extract
NMR	Nuclear magnetic resonance
PAS	Periodic acid-Schiff
PLS-DA	Partial least squares-discriminant analysis
STZ	Streptozotocin
